# Detection and subtyping avian metapneumovirus from turkeys in Iran 

**Published:** 2017-06-15

**Authors:** Mansour Mayahi, Hassan Momtaz, Ramezan Ali Jafari, Pejman Zamani

**Affiliations:** 1 *Department of Clinical Sciences, Faculty of Veterinary Medicine, Shahid Chamran University of Ahvaz, Ahvaz, Iran; *; 2 *Department of Veterinary Medicine, Veterinary Microbiology Section, Islamic Azad University of Shahrekord, Shahrekord, Iran;*; 3 *DVSc Student of Avian Health and Diseases, Department of Clinical Sciences, Faculty of Veterinary Medicine, Shahid Chamran University of Ahvaz, Ahvaz, Iran.*

**Keywords:** Avian metapneumovirus, Iran, RT-PCR, Turkey

## Abstract

Avian metapneumovirus (aMPV) causes diseases like rhinotracheitis in turkeys, swollen head syndrome in chickens and avian rhinotracheitis in other birds. Causing respiratory problems, aMPV adversely affects production and inflicts immense economic losses and mortalities, especially in turkey flocks. In recent years, several serological and molecular studies have been conducted on this virus, especially in poultry in Asia and Iran. The purpose of the present study was detecting and subtyping aMPV by reverse transcriptase polymerase chain reaction (RT-PCR) from non-vaccinated, commercial turkey flocks in Iran for the first time. Sixty three meat–type unvaccinated turkey flocks from several provinces of Iran were sampled in major turkey abattoirs. Samples were tested by RT-PCR for detecting and subtyping aMPV. The results showed that 26 samples from three flocks (4.10%) were positive for viral RNA and all of the viruses were found to be subtype B of aMPV. As a result, vaccination especially against subtype B of aMPV should be considered in turkey flocks in Iran to control aMPV infections.

## Introduction

Avian metapneumovirus (aMPV) can cause several diseases including turkey rhinotracheitis, swollen head syndrome in chickens and avian rhinotracheitis in other avian species. Secondary or co-infections with other organisms can deteriorate the situation.^1^ In addition to poultry, aMPV viral RNA has been detected in wild birds like house sparrows and ring-billed gulls by reverse transcriptase polymerase chain reaction (RT-PCR).^2^ The aMPV was identified for the first time in South Africa in turkey flocks in 1978.^3^ The aMPV infections lead to remarkable economic loss, especially in turkey flocks and are considered as an important disease in turkeys.^4^ Being highly contagious and acute, aMPV causes non-specific upper respiratory tract infections in turkeys.^5^ The aMPV is a negative sense RNA virus belonging to Paramyxoviridae family and stands in Pneumovirinae subfamily and genus Metapneumovirus.^6^ Four subtypes of this virus have been recognized: subtype A and B have been detected almost worldwide, especially in Europe, while subtype C has been found just in a few countries including USA, France and Korea and subtype D has been reported in France.^7^^-^^9^ An upper respiratory disease in human emerges by a genetically similar metapneumovirus called hMPV.^10^ Genetic studies have shown that hMPV emerged from subtype C of aMPV around 200 years ago.^11^ Due to non-specific clinical signs of aMPV infections, diagnosis is hard and also isolation of the virus is time consuming and difficult.^12^ The present study was designed for aMPV identification and subtyping in broiler turkey flocks in Iran. 

## Materials and Methods

**Sample collection. **Sampling was carried out between April 2014 and July 2015 in main turkey meat abattoirs in Iran. Sixty three non-vaccinated commercial broiler turkey flocks were examined in this study. Sterile Dacron swabs were used for choanal cleft sampling as advised in several studies.^12^^-^^16^ Before slaughtering, information of each flock was registered in flock working sheets and samples from choanal clefts of 10 birds of each flock aged 102 to 163 days were collected. A total of 630 samples were collected and examined. Swabs were dried in ambient temperature for 5 min, then, transferred to laboratory and stored at – 80 ˚C prior to RNA extraction.

**Extraction of RNA. **Each swab was diluted in 0.50 mL phosphate buffer saline and then, the extraction was implemented by RIBO-prep^®^ Nucleic acid extraction kit (Amplisens Co., Bratislava, Slovak Republic).

**Primers. **Detection and Subtyping were performed with reliable primers evaluated in several articles.^7^^,^^17^^,^^18^ The Nd/Nx andGa/Gy pair primers were utilized for screening the aMPV from all subtypes. The Ga/G2 pair was used for detecting subtype A, Ga/G12 for subtype B, G150/G1005 for subtype D and C1/C2 for subtype subtype C (Table 1). As a positive control, we used HIPRAVIAR^®^ SHS live vaccine subtype B of virus (Hipra Co., Gerona, Spain) and Nobilis TRT^®^ subtype A (Intervet, Boxmeer, The Netherlands). The Bronhikal^®^ І SPF (Genera Co., Žitarka, Croatia) H120 strain of infectious bronchitis live vaccine was used as a negative control (Table 1).

**Table 1:** Primer sequences used for qRT-PCR.

**Primer**	**Gene**	**Primer’s Sequence**	**Ref.**
**Ga(forward)**	G	5’- CCGGGACAAGTATCTCTATGG -3’	17
**Gy(reverse)**	G	5’- TCTCGCTGACAAATTGGTCCTGA -3’	17
**Nd(forward)**	N	5’- AGCAGGATGGAGAGCCTCTTTG -3’	17
**Nx(Reverse)**	N	5’- CATGGCCCAACATTATGTT -3’	17
**G2(forward)**	G	5’- CCACACTTGAAAGATCTACCC -3’	17
**G12(reverse)**	G	5’- CAGTCGCCTGTAATCTTCTAGGG -3’	17
**C1(forward)**	M	5’-- GATGACTACAGCAAACTAGAG -3’	19
**C2(reverse)**	M	5’- CTTCAGGACATATCTCGTTAC -3’	19
**G150(forward)**	G	5’- CCGATGCCCAGTTAATAA -3’	7
**G1005(reverse)**	G	5’- CCCCTTACAAACACTGTTC -3’	7

**The cDNA synthesizing procedure. **The cDNA was synthesized by RevertAid^®^ first strand cDNA synthesis kit (Thermo Fisher Scientific Inc., Vilnius, Lithuania). The total volume of the mix in RNASE-free microtubes was reached 20 μL. The PCR amplification of first strand cDNA was performed in a thermal cycler (Labgene Scientific Co., Zurich, Switzerland).

**The PCR amplification. **The PCR was performed with 10 μL Parstous PCR Mix^®^ (Pars-Tous, Mashhad, Iran), 2 μL forward and reverse primers, 5 μL DEPC water (CinnaGen, Karaj, Iran) and 3 μL cDNA of samples. The total volume of PCR Mix was 20 μL. The following procedure was used for amplification: 15 min at 45 ˚C for 1 cycle as pre-cycle, followed by 30 cycles of 20 sec at 94 ˚C, annealing temperature based on primer pairs for 45 sec (Table 2), 45 sec at 72 ˚C and a final extension of 10 min at 72 ˚C. The PCR products were visualized by DNA safe stain (CinnaGen) on 1.50% agarose gel that had been electrophoresed at 140 V for 40 min (Table 2).

**Table 2:** Primer sets specifications used for detection and subtyping of aMPV in this study, annealing temperatures and RT-PCR product sizes

**Gene **	**Primer set**	**aMPV subtype**	**Annealing temperature (**˚C**)**	**Product size (bp)**
**N**	Nd/Nx	All	51	115
**G**	Ga/Gy	All	54	448
**G**	Ga/G2	A	54	504
**G**	Ga/G12	B	54	312
**M**	C1/C2	C	51	468
**G**	G150/G1005	D	57	956

## Results

**Fig. 1:**
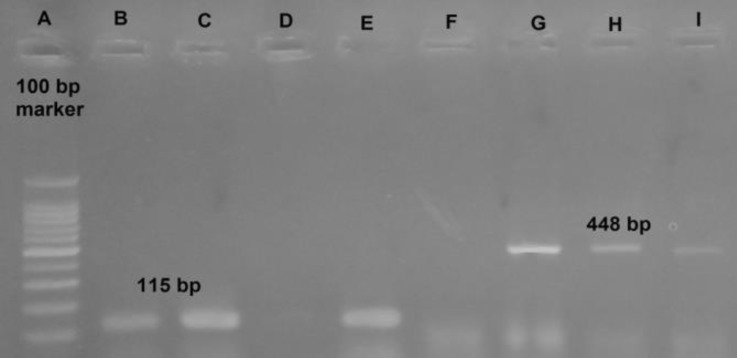
A: Ladder, B and C: Positive samples, D: Negative control, E: Positive control, F: Negative control, G: Positive control, H and I: Positive samples, (B to E are Nd/Nx primer’s products and F to I are Ga/Gy products

The swabs (n = 630) out of 63 flocks were obtained (10 swabs from each flock). Twenty*-*six samples out of three flocks were positive (the numbers of positive swabs were 8, 8 and 10 swabs out of each of the three flocks). All of these three flocks had experienced some upper respiratory problems during their rearing periods. No difference was found in detection rate of Nd/Nx and Ga/Gy primers as screening primers (the size of PCR product for Nd/Nx pair primers was 115 bp and 448 bp for Ga/Gy). All positive samples detected by subtyping primers were identified as subtype B viruses. The RNA of aMPV subtype B was detected in 4.10% of all samples (Fig. 1). 

## Discussion

Several serological studies have been done in Iran; 56.52% of 182 blood samples from 18 broiler chick flocks collected from slaughterhouses were positive for aMPV antibodies by enzyme-linked immunosorbent assay (ELISA) though none of these flocks had been vaccinated against aMPV.^19^ It was found that 93.20% of 88 samples from broiler parent stocks and 48.10% of broiler samples were serologically positive for aMPV.^20^ Seroprevalence of aMPV in broiler turkeys in Iran by ELISA method has been investigated and 60.00% of 200 blood samples from seven flocks were positive.^21^ The aMPV has been detected in broilers by nested RT-PCR in Ahvaz, Iran. It was found that 28.00% of all samples had aMPV viral RNA.^13^ Previously, 43 broilers, five layers and two broiler parent stocks with clinical respiratory signs have been inspected for aMPV by RT-PCR. Sixteen percent of all flocks were found positive for aMPV viral genome.^22^ Prevalence of aMPV subtypes by RT-PCR in broiler turkeys at a local abattoir has been investigated^16^ and with this molecular technique, only one clinically healthy flock out of 23 was positive. The results of this study are very similar to those of ours. The results of both studies suggest that in spite of high serological evidence for aMPV, the virus propagates in a very short period of time in bird bodies. Another point is that detection rate can be higher in early ages rather than older ages at the end of rearing period. Therefore, we suggest aMPV molecular detection in flocks which show upper respiratory problems at early ages. 

The RT-PCR is a powerful laboratory detection tool for aMPV viral RNA, but serological methods are beneficial for screening and monitoring turkey flocks in large scales. Based on our study and others, subtype B seems to be the dominant aMPV subtype in Iran and also in the Middle East.^16^^,^^18^^,^^22^ We suggest that more molecular studies be conducted with special attention to those that have sequencing of existed strains in Iran, especially on turkeys with clinical signs. Such studies will definitely help us make better vaccines and design more powerful vaccination strategies. Also, we recommend more investigations in other types of poultry and wild birds to find out if other known subtypes of aMPV such as subtype C and A ,which have already been detected in Asia, exist or not. Detecting subtype D is of less priority than other subtypes because it was found just one time in France about two decades ago and never detected again.^7^^,^^23^^,^^24^ In addition, we suggest more serological monitoring studies to find out how big the aMPV problem is in Iranian poultry industry. In conclusion, subtype B is the dominant aMPV subtype found in Iran, making turkey flock vaccination necessary to control the infections.
